# FTY720 Reduces Post-Ischemic Brain Lymphocyte Influx but Does Not Improve Outcome in Permanent Murine Cerebral Ischemia

**DOI:** 10.1371/journal.pone.0021312

**Published:** 2011-06-20

**Authors:** Arthur Liesz, Li Sun, Wei Zhou, Sönke Schwarting, Eva Mracsko, Markus Zorn, Henrike Bauer, Clemens Sommer, Roland Veltkamp

**Affiliations:** 1 Department of Neurology, University Heidelberg, Heidelberg, Germany; 2 Department of Internal Medicine, University Heidelberg, Heidelberg, Germany; 3 Department of Neuropathology, University Medical Center Mainz, Mainz, Germany; National Institute of Allergy and Infectious Diseases - Rocky Mountain Laboratories, United States of America

## Abstract

**Background:**

The contribution of neuroinflammation and specifically brain lymphocyte invasion is increasingly recognised as a substantial pathophysiological mechanism after stroke. FTY720 is a potent treatment for primary neuroinflammatory diseases by inhibiting lymphocyte circulation and brain immigration. Previous studies using transient focal ischemia models showed a protective effect of FTY720 but did only partially characterize the involved pathways. We tested the neuroprotective properties of FTY720 in permanent and transient cortical ischemia and analyzed the underlying neuroimmunological mechanisms.

**Methodology/Principal Findings:**

FTY720 treatment resulted in substantial reduction of circulating lymphocytes while blood monocyte counts were significantly increased. The number of histologically and flow cytometrically analyzed brain invading T- and B lymphocytes was significantly reduced in FTY720 treated mice. However, despite testing a variety of treatment protocols, infarct volume and behavioural dysfunction were not reduced 7d after permanent occlusion of the distal middle cerebral artery (MCAO). Additionally, we did not measure a significant reduction in infarct volume at 24h after 60 min filament-induced MCAO, and did not see differences in brain edema between PBS and FTY720 treatment. Analysis of brain cytokine expression revealed complex effects of FTY720 on postischemic neuroinflammation comprising a substantial reduction of delayed proinflammatory cytokine expression at 3d but an early increase of IL-1β and IFN-γ at 24 h after MCAO. Also, serum cytokine levels of IL-6 and TNF-α were increased in FTY720 treated animals compared to controls.

**Conclusions/Significance:**

In the present study we were able to detect a reduction of lymphocyte brain invasion by FTY720 but could not achieve a significant reduction of infarct volumes and behavioural dysfunction. This lack of neuroprotection despite effective lymphopenia might be attributed to a divergent impact of FTY720 on cytokine expression and possible activation of innate immune cells after brain ischemia.

## Introduction

Ischemic stroke is a major cause of death and disability worldwide. Currently, the only approved therapy for acute stroke is rapid vascular recanalization which restores the supply of blood to ischemic tissue [1]. Beyond a shortage of essential metabolites, ischemia triggers many other detrimental cascades. In particular, inflammatory mechanisms have come into the focus of research because they contribute substantially to secondary brain damage [2], [3] and their prolonged kinetics makes them amenable to therapeutic intervention [4], [5]. Recent experimental studies suggest a pivotal role of T cells in post-ischemic inflammation of various organs including the brain [6], [7], [8], [9], [10]. Although T lymphocytes are now known to patrol the CNS, the presence and activity of systemic immune cells in the healthy brain remains very restricted and tightly controlled by the intact blood-brain-barrier [11]. Cerebral invasion by inflammatory cells is a hallmark in the pathogenesis of neuroinflammation and contributes substantially to secondary tissue loss in acute stroke [12], [13], [14]. Similar to primary inflammatory CNS disease, deleterious T cell effector mechanisms in the ischemic brain include proinflammatory cytokines and potentially direct cytotoxicity [15], [16]. Consequently, preventing the CNS invasion of lymphocytes after brain ischemia may be a potent strategy for stroke therapy.

Fingolimod (FTY720) has emerged over the last few years as a potent treatment for clinical multiple sclerosis [17], [18], [19]. FTY720 is rapidly phosphorylated after administration and resemles thereby the structure of sphingosine-1-phosphate (S1P). Phosphorylated fingolimod binds to S1P receptors which are required for the emigration of extravascular leukocytes from tissues [20]. By functionally antagonizing the S1P receptors, FTY can modulate the migration of leukocytes. In previous studies in primary neuroinflammatory disease models, FTY720 effectively inhibited lymphocyte immigration into the CNS, dampened the humoral immune respone, and improved the functional outcome after experimental autoimmune encephalomylitis [21], [22], [23], [24].

Recently, the effectiveness of FTY720 was also tested in murine models of ischemic brain injury [25], [26], [27], [28]. These studies showed a beneficial effect of FTY720 on stroke outcome in a model of transient ischemia in mice [25], [26], [28] and rats [27]. However, the majority of reports studied the effect on infarct volume only at early time points (24 h to 4d after MCAO) when the postischemic invasion of lymphocytes, the key targets of FTY720, has just begun. Indeed, the mechanisms underlying the protective effects of FTY were only partially characterized. Surprisingly, none of the previous studies which were suggesting a neuroprotective role of FTY720 in stroke has analyzed the number of invading lymphocytes into the brain after stroke. Finally, all three studies used a experimental ischemia model that induced extensive lesions of approx. 50% of the hemisphere – a model which is known to be associated with high mortality, induces substantial changes in the systemic immune system and reflects only a small proportion of clinical strokes in patients [29], [30].

We therefore re-investigated the impact of FTY720 on stroke outcome in two experimental mouse models inducing moderate cortical lesions or extensive lesions and analyzed the modulation of neuroinflammatory effector mechanisms after brain ischemia.

## Results

### FTY720 reduces the number of circulating leukocytes

**Figure 1:**
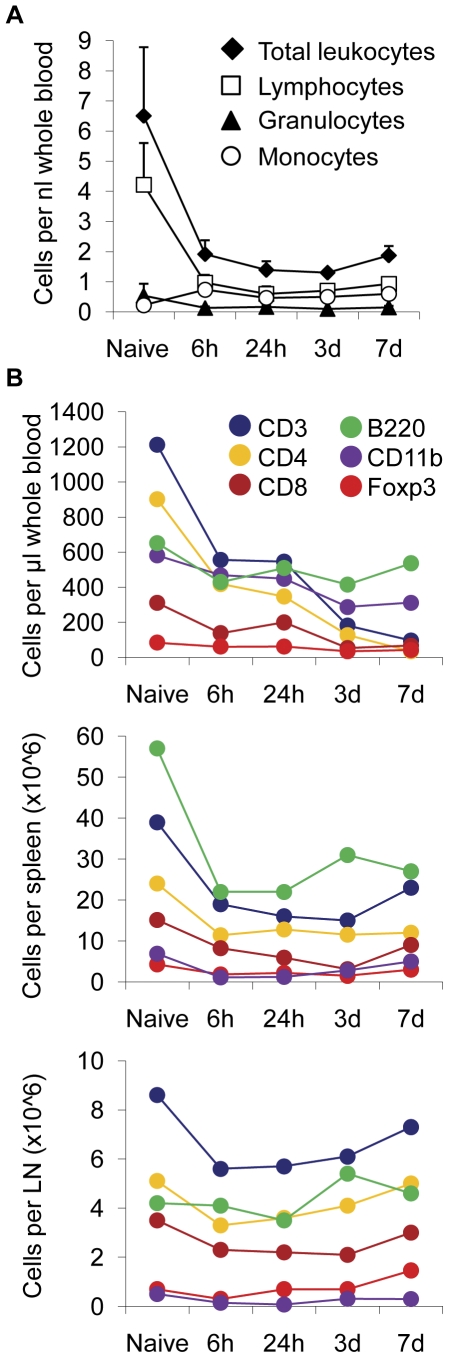
FTY720 treatment induces leukopenia. (**A**) Differential blood cell counting was performed in normal mice (Naive) and 6 h, 24 h, 3d and 7d after daily administration of FTY720. Mean values (n = 5 per time point) are depicted for total leukocytes, granulocytes, lymphocytes and monocytes as cells per µl whole blood. (**B**) Leukocyte subpopulations were further characterized by specific epitope markers for T cells (CD3, CD4, CD8), B cells (B220), regulatory T cells (Foxp3) and monocytes (CD11b) at the indicated time points in blood, spleen and mesenteric lymph nodes (n = 5 per group). Each experiment was performed 2–3 times.

We analyzed the cell number of leukocytes and lymphocyte subsets in blood and immunological organs to verify the putative lymphocyte arrest in secondary lymphatic organs by FTY720 treatment. Differential blood cell counts after daily administration (1 mg/kg body weight per day) of FTY720 by oral gavage revealed a substantial reduction of circulating leukocytes (**Fig. 1A**). This effect was mainly due to the decrease in the lymphocyte population by >80% as early as 6 h after the first dose of FTY720. Lymphocyte counts remained significantly reduced up to 7d of FTY720 treatment. The number of circulating granulocytes was unchanged after FTY720 treatment whereas the initially very low number of circulating monocytes was increased about 3-fold already 6 h after FTY720 treatment (before: 220±110 cells/µl, 6 h: 730±230 cells/µl).

Further analysis of lymphocyte subpopulations revealed that mainly CD4+ T cells were reduced in blood after FTY720 treatment, while other lymphocytes remained largely unaffected (**Fig. 1B** **and** **Table 1**). Similar to the pattern found in blood, a significant reduction of T cells and also B220+ B cells was found in spleens after FTY720 administration (**Fig. 1B** **and** **Table 1**). In contrast, the number of lymphocytes in mesenteric lymph nodes was not significantly affected at any time point after FTY720 treatment while CD11b cells were significantly reduced at 6 h and 24 h after FTY720 administration (**Fig. 1B** **and** **Table 1**).

**Table 1:** Mean values and standard deviations of data shown in Figure 1b.

	CD3	CD4	CD8	Foxp3	B220	CD11b
**Blood**						
Naïve	1,213±165	902±117	311±77	84±25	651±181	582±86
6 h	* 557±98	* 419±51	* 138±53	62±29	* 430±91	469±302
24 h	* 546±123	* 347±216	200±166	62±27	510±210	449±180
3d	* 182±41	* 182±41	* 54±14	* 35±26	416±219	* 288±140
7d	* 97±28	* 35±9	* 68±17	* 42±16	537±175	* 312±91
**Spleen**						
Naïve	39.4±8.4	24.3±7.7	15.3±6.1	4.2±2.0	57.7±14.4	6.9±1.8
6 h	* 19.6±5.8	* 11.4±3.5	* 8.2±2.5	* 1.8±0.5	* 21.8±5.1	* 1.1±0.3
24 h	* 15.9±6.0	* 12.8±4.6	* 5.9±2.2	* 2.1±0.5	* 22.4±6.4	* 1.2±0.2
3d	* 14.5±6.7	* 11.5±6.7	* 3.1±1.3	* 1.5±0.8	* 31.0±6.3	* 2.8±1.0
7d	* 23±9	* 12.1±4.9	* 9.0±2.3	3.2±0.7	* 27.1±5.3	4.8±1.6
**Lymph node**						
Naïve	8.6±2.9	5.1±1.7	3.5±0.8	0.7±0.2	4.3±1.5	0.5±0.2
6 h	5.6±1.6	3.3±1.2	2.3±0.9	0.4±0.3	4.0±1.7	* 0.2±0.06
24 h	5.8±0.9	3.6±0.7	2.2±0.7	0.7±0.2	3.5±0.8	* 0.1±0.02
3d	6.1±1.5	4.1±0.9	2.1±0.7	0.7±0.1	5.4±1.3	0.3±0.3
7d	7.3±1.5	5.0±1.2	3.2±1.4	1.5±0.4	4.6±1.0	0.3±0.2

* = p<0.05 between Naïve mice and respective time point after FTY720 (n = 5 per group).

### FTY720 treatment does not improve outcome after experimental ischemic stroke

**Figure 2:**
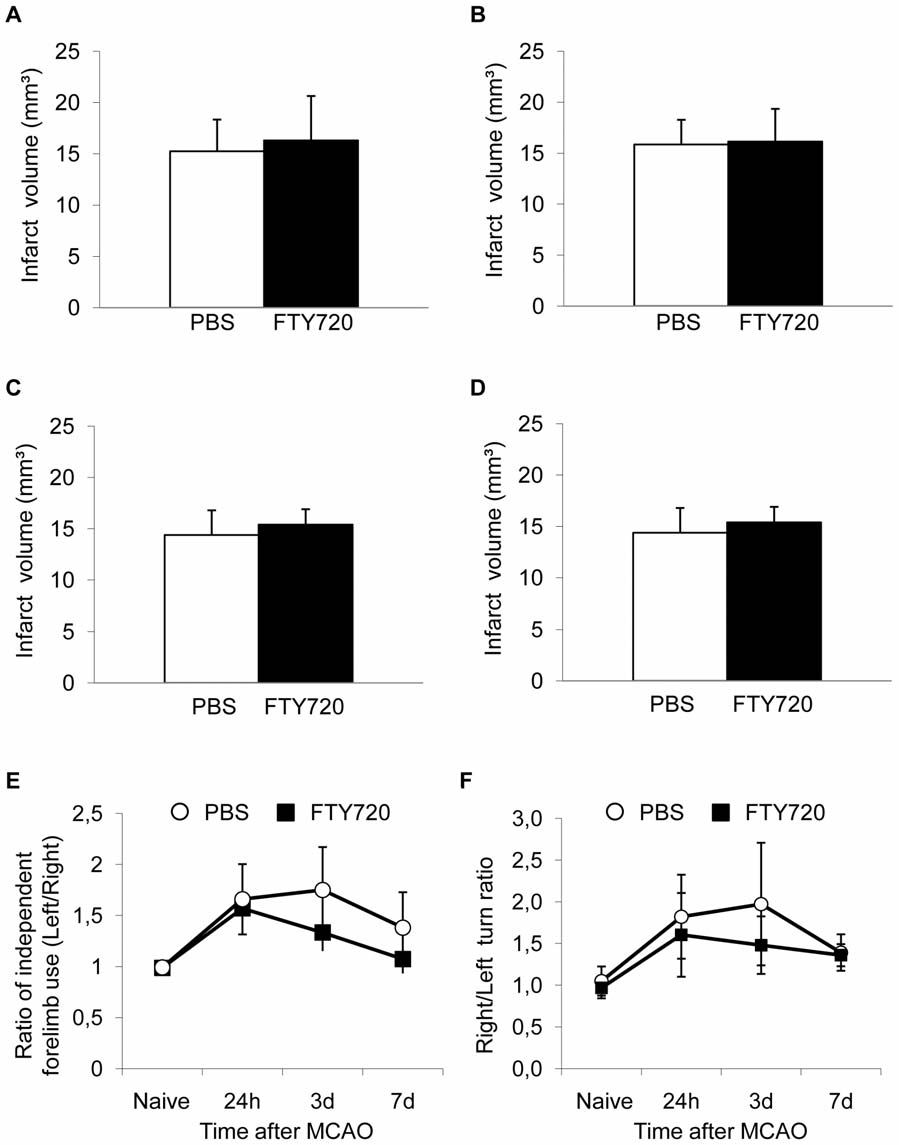
FTY720 does not reduce infarct volume after cortical permanent ischemia. Animals in the treatment groups (FTY720) received daily administrations of 1 mg/kg FTY720 by oral gavage starting at 48 h before infarct induction.I Infarct volumes were determined (**A**) at 3d (n = 8 per group, p = 0.73, 2 individual experiments) and (**B**) at 7d (n = 17, 0.54, 4 individual experiments) after infarct induction. Control animals received daily PBS injections. (**C**) Animals were treated daily with either FTY720 or PBS, starting from 3 h after MCAO and infarct volumes were determined at 7d after brain ischemia (n = 10, p = 0.43, 2 individual experiments). (**D**) Mice received a single dose of FTY720 or PBS at 48 h before brain ischemia and infarct volumetry was performed at day 7 (n = 10, p = 0.27). Behavioural dysfunction and recovery after experimental stroke was assessed in FTY720 pretreated animals (daily treatment starting 48 h before MCAO) or in control animals by the (**E**) “cylinder test” (n = 12, 3 individual experiments) and the (**F**) “corner test” (n = 12, 3 individual experiments).

Previous studies [25], [26], [27] have shown a neuroprotective effect of FTY720 on stroke outcome in reversible middle cerebral artery occlusion (MCAO) models. We analyzed the effect of daily FTY720 treatment on infarct volume and behavioural dysfunction after permanent coagulation of the MCA distal of the lenticulostriatal arteries. This permanent ischemia model induces moderately sized cortical infarct lesions. We analyzed infarct volumes in mice receiving daily administrations of 1 mg/kg FTY720 by oral gavage compared to PBS treated control animals in a pre-treatment protocol starting 48 h before MCAO and in a post-treatment protocol starting the treatment 3 h after MCAO. We did not detect a significant difference of infarct volumes 3d and 7d after MCAO in the pre-treatment protocol starting 48 h before MCAO (**Fig. 2A,B**). Infarct volumes were also not significantly reduced at 7d after MCAO in animals receiving FTY720 by the post-treatment protocol starting 3 h after MCAO (**Fig. 2C**). We additionally analyzed the effect of a single injection of FTY720 to exclude the possibility of cumulative dose toxicity by the daily treatment, but did also not measure a significant reduction in infarct volume at 7d after MCAO in animals that were treated with a single dose of FTY720 or PBS at 48 h before MCAO. Moreover, we tested the postischemic sensorimotor function by the “Corner test” [31] and the “forelimb use asymmetry test” [32] which both correlated well with the infarct volume within the first week after MCAO in a previous study [13]. We did not detect a significant reduction of behavioural dysfunction by FTY720 compared to controls at any measured time point after MCAO in both tests (**Fig. 2E,F**).

**Figure 3:**
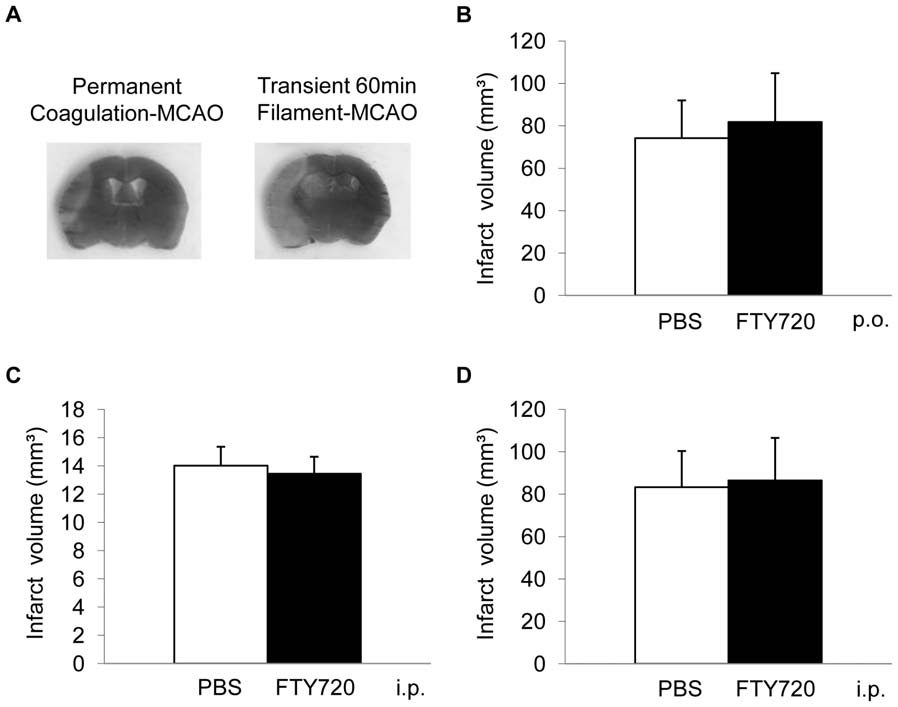
FTY720 does not alter infarct volume in alternative experimental protocols. (**A**) Representative images of brain sections stained by the silver-staining technique from coagulation-MCAO and transient 60 min filament-MCAO as used for determination of infarct volumes. (**B**) Brain ischemia was induced by 60 min reversible MCAO and infarct volumes were determined at 24 h after ischemia in animals receiving daily oral administration of FTY720 or PBS starting 48 h before stroke induction (n = 12, p = 0.42, 3 individual experiments). (**C, D**) FTY720 or PBS were administered by daily i.p. injection starting 48 h berfore MCAO. (**C**) MCAO was induced by transcranial permanent coagulation and infarct columes determined at 7d after MCAO (n = 10, p = 0.36, 2 individual experiments). (**D**) MCAO was induced by transient 60 min filament-occlusion of the MCA and infarct volume measured at 24 h after MCAO (n = 9, p = 0.86, 2 individual experiments).

**Figure 4:**
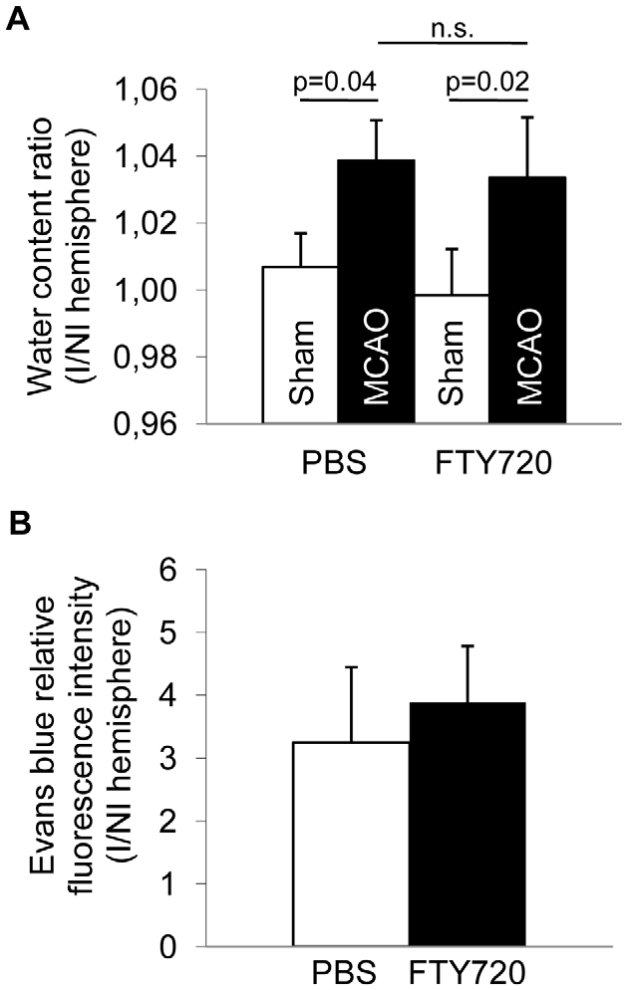
FTY720 does not influence brain edema and blood-brain-barrier permeability. (**A**) Brain water content was analyzed as the percentage water content per hemisphere by the “wet/dry weight” method and is shown as the water content ratio of ischemic/non-ischemic hemispheres. Open bars represent water content ratios in animals at 3d after Sham operation (“Sham”) and black bars in mice at 3d after permanent coagulation MCAO (“MCAO”) receiving FTY720 or PBS treatment by oral gavage (n = 5 per group, 2 individual experiments). (**B**) Blood-brain-barrier permeability was tested using the Evans blue assay for dye extravasation at 3d after permanent coagulation MCAO in mice receiving FTY720 or PBS treatment by oral gavage. Data is shown as the ratio for ischemic/non-ischemic hemisphere fluorescence intensity at 680 nm (n = 5, one experiment).

To test the hypothesis of a differential effect of *per os* FTY720 treatment in an infarct model of ischemia-reperfusion injury of the brain in contrast to permanent MCA occlusion, we analyzed infarct volumes 24 h after MCAO in 48 h-pretreated animals undergoing 60 min reversible occlusion of the MCA (**Fig. 3B**). We did not detect a significant effect of FTY720 treatment compared to PBS on the resulting infarct volume. Additionally, we tested whether intraperitoneal (i.p.) administration of FTY720 would have a neuroprotective effect in contrast to administration by oral gavage. Neither in at 7d after MCAO in the coagulation model (**Fig. 3C**) nor at 24 h after MCAO in the 60 min filament-occlusion model (**Fig. 3D**) a significant difference between FTY720 and PBS treated animals was detected. Furthermore we analyzed brain edema formation as a marker of blood-brain-barrier dysfunction in normal animals receiving FTY720 or PBS and in mice at 3d after MCAO in both treatment groups (**Fig. 4A**). We detected a significant increase in brain water content of the ischemic hemisphere at 3d after MCAO compared to brains 3d after Sham operation, but we did not measure a difference between edema in PBS treated and FTY720 treated animals (**Fig. 4A**). Additionally, blood-brain-barrier permeability was analyzed by measuring Evans blue fluorescence intensity in brain homogenates at 3d after permanent MCAO in FTY720 treated mice and controls. We did not measure a significant difference in the ratio of ischemic/non-ischemic hemispheres for the Evans blue fluorescence intensity (**Fig. 4B**).

### Physiological cardiovascular parameters are not affected by FTY720 treatment

**Figure 5:**
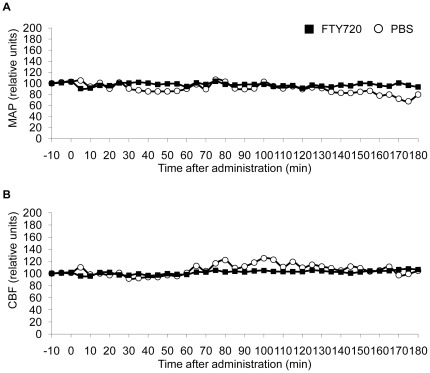
FTY720 does not affect basal cardiovascular parameters. Mean arterial pressure (**A**) and cerebral blood flow (**B**) were recorded for 10 min before and 180 min after oral administration of 1 mg/kg FTY720 in 100 µl PBS or 100 µl PBS alone. Values are expressed as relative units in relation to baseline values before the respective treatment (n = 3 per group, each mouse was an individual experiment).

We investigated the effect of FTY720 on basal physiological parameters to exclude major cardiovascular side effects of the treatment. Therefore, we continuously measured mean intra-arterial pressure (MAP) after catherization of the femoral artery (**Fig. 5A**) and relative cerebral blood flow by laser doppler analysis (**Fig. 5B**) for 10 min before and 180 min after oral FTY720 administration. We did not detect a significant alteration of those parameters between treatment groups.

### FTY720 treatment decreases cerebral lymphocyte invasion

**Figure 6:**
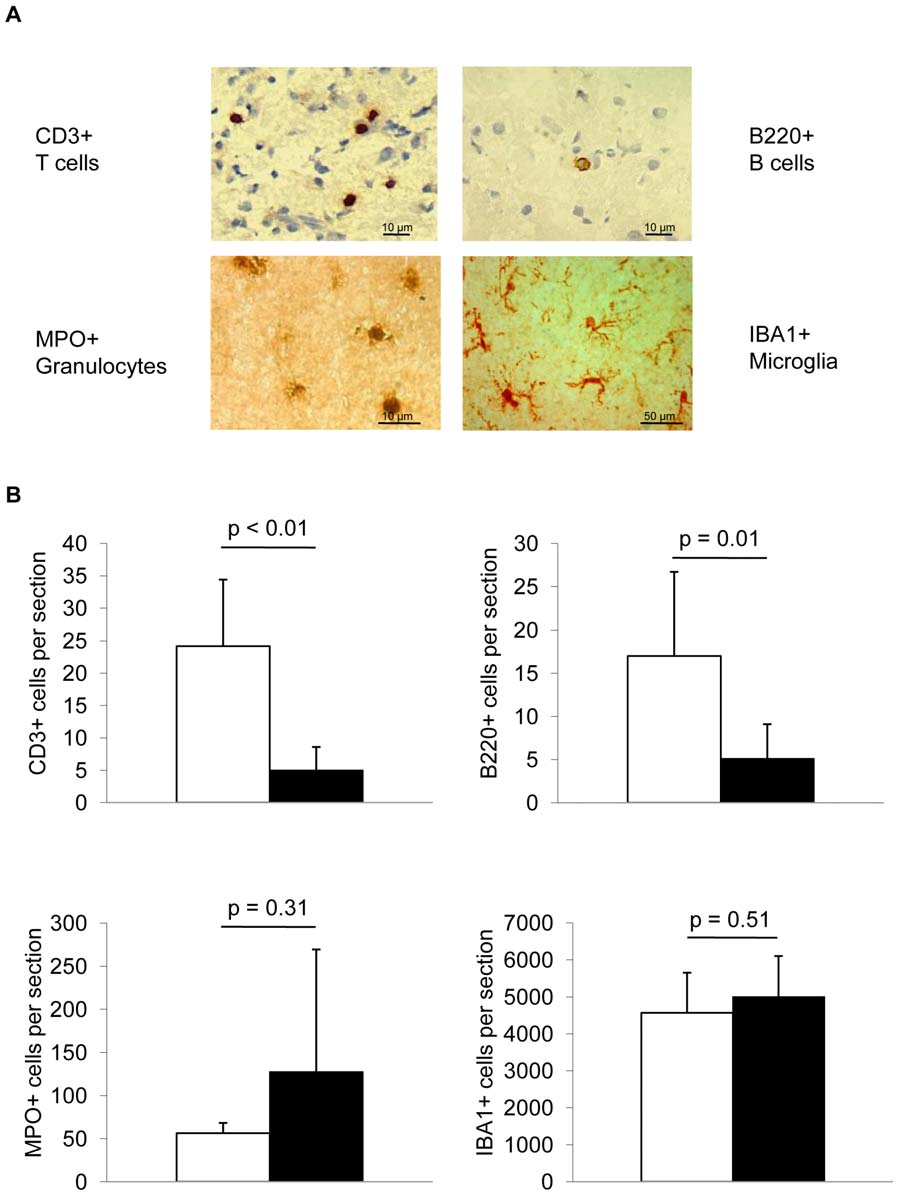
FTY720 decreases cerebral lymphocyte invasion. (**A**) Brain sections were stained 5d after MCAO for T cells (CD3), B cells (B220), granulocytes (MPO) and activated microglia/macrophages (IBA1). (**B**) Analysis of absolute cell counts of T cells, B cells, granulocytes and microglia/macrophages per total hemisphere in PBS and FTY720 treated animals at 5d after MCAO (n = 6–10 per group, 2–3 individual experiments).

We performed histological analysis for the invasion of systemic leukocytes and for activation of resident microglial cells to test the effect of FTY720 on cellular inflammation after experimental brain ischemia (**Fig. 6**). We stained histological sections at 5d after MCAO in FTY720 treated mice (1 mg/kg, daily treatment, starting 48 h prior to MCAO) and control animals for the expression of CD3 (T cells), B220 (B cells), MPO (granulocytes) and IBA1 (microglia/macrophages) (**Fig. 6A**). FTY720 treatment significantly reduced cerebral invasion of T cells and B cells compared to control animals (**Fig. 6B**). However, MPO-positive cells (i.e. mainly neutrophil granulocytes) did not significantly differ between treatment groups. Individual animals in the FTY720 treated group had substantially increased MPO+ cell counts (**Fig. 6B**). No significant difference was detectable for the expression of IBA+ cells in brain sections of FTY720 or PBS treated animals, a marker for activated microglia/macrophages [33]. We additionally performed flow cytometric analysis of immune cell subpopulations isolated from brains of PBS- or FTY720-treated animals 5d after MCAO to confirm histological data (**Table 2**) by a previously reported FACS gating strategy [34]. Correspondingly, we observed a substantial reduction of brain invading T-helper (CD3+/CD4+) and T-effector (CD3+/CD8+) cells in FTY720 treated animals compared to controls. Absolute cell counts per ischemic hemisphere were not significantly different for Granulocytes (CD45+/Gr-1+), NK cells (CD45+/NK1.1+) and for antigen-presenting cells (CD11b+/MHC-II+) between the treatment groups (**Table 2**).

**Table 2:** Flow cytometric analysis of brain immune cells at 5d after permanent MCAO.

	CD3/CD4	CD3/CD8	CD45/Gr-1	CD45/NK1.1	CD11b/MHC-II
PBS	5067±2349	9100±6930	57333±27341	49000±43841	17033±10866
FTY720	2303±1842	3833±1746	54000±59397	29000±32527	10033±11832
p value	0.091	0.039	0.875	0.274	0.326

Cells per one hemisphere (Mean±SD). N = 6 mice per group. Data of 2 individual experiments.

### FTY720 alters cerebral cytokine expression

**Figure 7:**
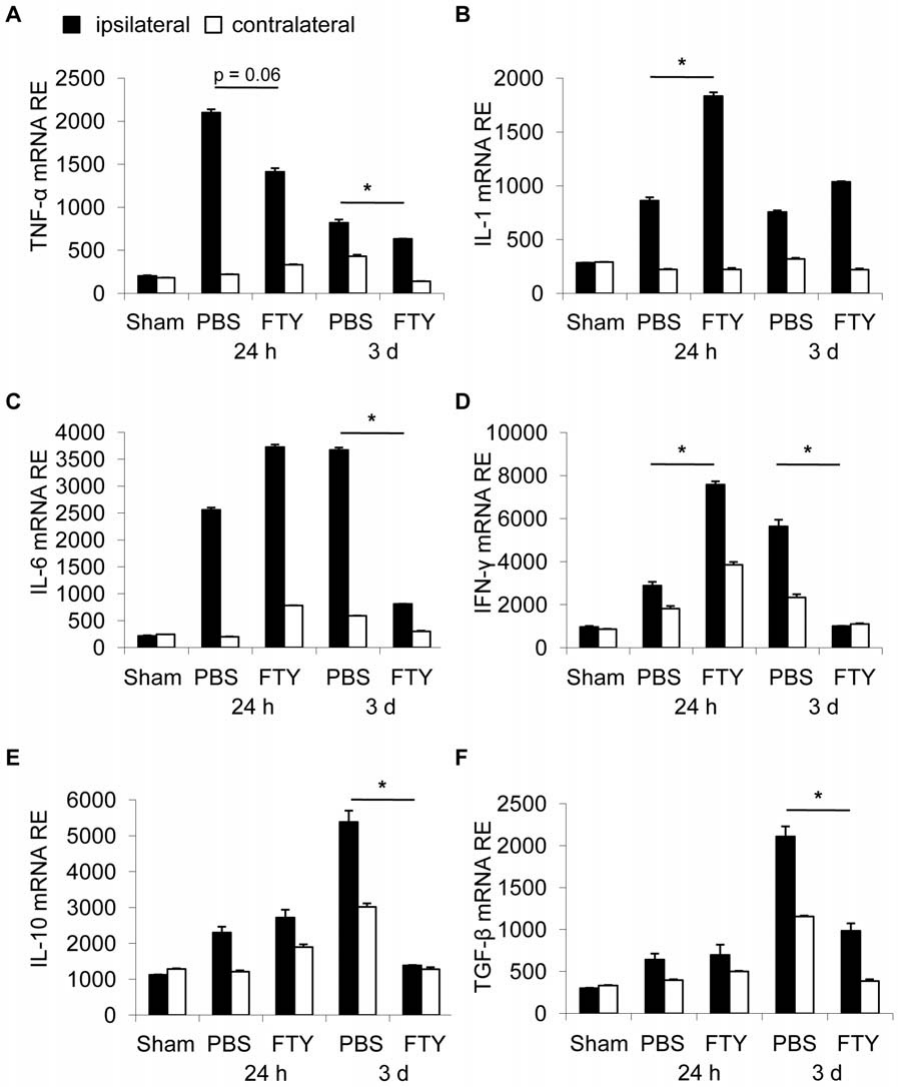
FTY720 treatment affects post-ischemic cerebral cytokine expression. Cytokine mRNA relative expression (RE, normalized to Ppia housekeeping gene) for pro-inflammatory (TNF-α, IL-1β, IL-6 and INF-γ, **A-D**) and anti-inflammatory (IL-10 and TGF-β, **E,F**) cytokines was measured at 24 h and 3d after MCAO by RT-PCR in the ischemic (black bars) and nonischemic (open bars) hemispheres of control (PBS) and FTY720 treated mice (n = 8 per group, 2–3 individual experiments per group). * P<0.05 between ischemic hemispheres of treatment and control groups at one respective time point.

To further address the influence of FTY720 on post-ischemic neuroinflammation, we measured cerebral cytokine expression in ischemic and non-ischemic hemispheres at 24 h and 3d after MCAO in PBS and FTY720 treated animals (1 mg/kg, daily p.o. treatment, starting 48 h prior to MCAO). We analyzed the expression of the proinflammatory cytokines TNF-α, IL-1β, IFN-γ and IL-6 (**Fig. 7A-D**) as well as the anti-inflammatory cytokines IL-10 and TGF-β (**Fig. 7E,F**). At 3d after MCAO we detected a significant reduction of all measured cytokines in FTY720 treated animals compared to control mice except for IL-1β. Interestingly, at 24 h after MCAO the expression pattern was partially inverse with a significant increase in the expression of IL-1β (**Fig. 7B**) and IFN-γ (**Fig. 7D**) in the FTY720 group compared to control animals [13], [29].

### FTY720 differentially affects systemic cytokine secretion

**Figure 8:**
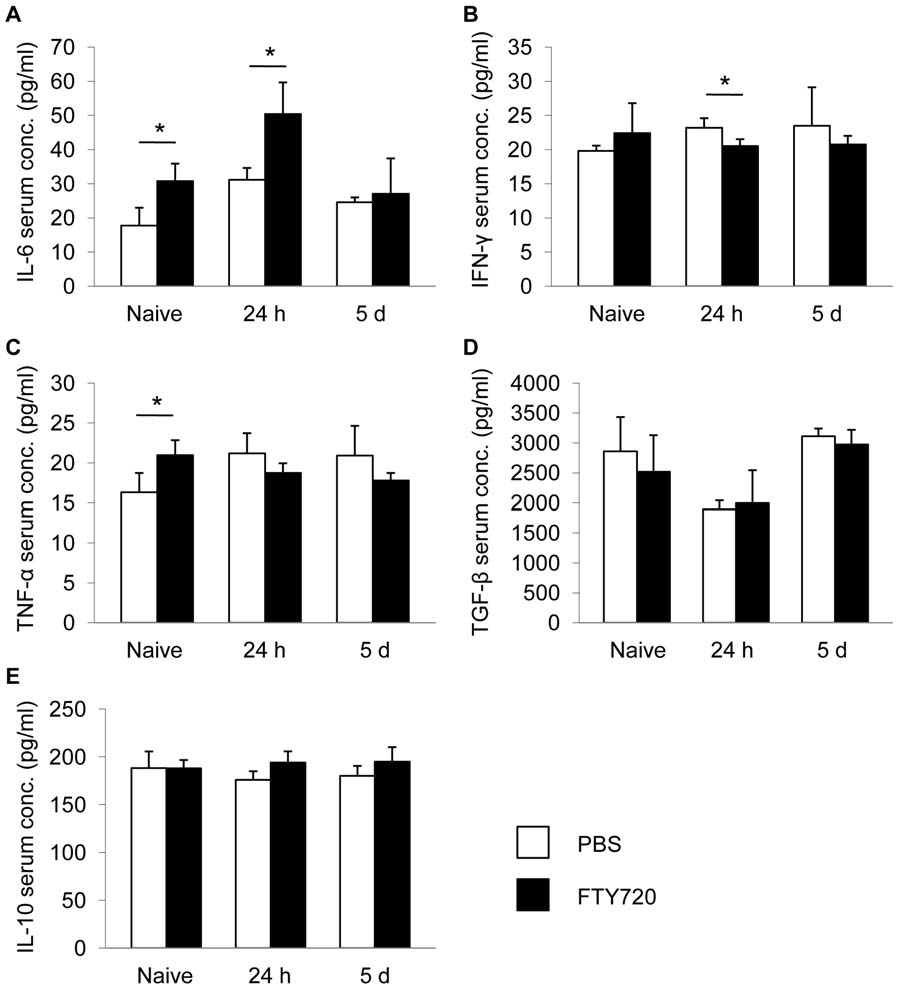
FTY720 changes serum cytokine levels. Serum cytokine concentrations of the pro-inflammatory cytokines IL-6, IFN-γ and TNF-α (**A–C**) and anti-inflammatory cytokines TGF-β and IL-10 (**D,E**) were measured in naïve mice and at 24 h and 5d after FTY720 or control treatment. Each assay was performed in duplicate (n = 5, serum sampling as 2–3 individual experiments, assays as one experiment). * P<0.05 between treatment groups at the respective time point.

We also analyzed serum cytokine levels in naïve animals and at 24 h and 5d after MCAO in PBS- and FTY720-treated animals to control for systemic humoral alterations. We measured cytokine concentrations of the pro-inflammatory cytokines IL-6, IFN-γ and TNF-α (**Fig. 8A-C**) as well as the anti-inflammatory cytokines TGF-β and IL-10 (**Fig. 8D,E**). Interestingly, we detected a significant increase of serum concentrations of IL-6 and TNF-α in naïve animals after FTY720 treatment compared to control animals (**Fig. 8A,C**). At 24 h after MCAO we also measured an increase of IL-6 levels (**Fig. 8A**) but a reduction of IFN-γ concentrations in FTY720 treated animals (**Fig. 8B**). Cytokine levels of the anti-inflammatory cytokines TGF-β and IL-10 were not altered by the treatment (**Fig. 8D,E**). Taken together, these results might indicate a partial early activation of the systemic immune system by FTY720 administration.

## Discussion

This preclinical study was conducted to evaluate the potential of FTY720 for the treatment of ischemic stroke. The main findings of this study are that 1) FTY720 reduces postischemic cerebral lymphocyte influx but 2) does not improve outcome after focal ischemic stroke after permanent focal ischemia 3) FTY treatment had complex and partially opposite effects on cerebral and systemic inflammatory markers which may explain the lack of efficacy on outcome after experimental stroke.

Previous studies analyzed infarct outcome 24 h or 4d after stroke in mice and up to 3d after brain ischemia in rats, respectively [25], [26], [27]. Interestingly, the protective effects in these studies are unlikely to have been caused by prevention of postischemic brain invasion of lymphocytes – the main mechanism of action of FTY720 in multiple sclerosis and experimental autoimmune encephalitis [18], [20], [22], [35]. Invasion of leukocytes into the brain takes place in a distinct kinetic order for each subpopulation. While the invasion of monocytes/macrophages and granulocytes can take place within hours after brain ischemia, relevant numbers of lymphocytes begin to invade the brain only 3–5 days after experimental stroke [13], [25], [36], [37], [38]. Therefore, other mechanisms than inhibition of brain lymphocyte influx would be needed to explain the observed effects. In a recent report by Wei and coworkers a prolonged beneficial effect of FTY720 up to 14d after ischemia was shown [28]. Interestingly, FTY improved the behavioural dysfunction while infarct volume, neuronal apoptosis and inflammatory markers were not affected and cerebral lymphocyte immigration was not examined. As *in vitro* experiments did not support anti-apoptic effects on neurons, the mechanisms underlying the protective action of FTY720 *in vivo* remained unclear [28].

All of the previous studies investigating the effect of FTY720 on infarct outcome used an experimental stroke model that differs from the model used for the majority of experiments in the present study [25], [26], [27], [28]. In contrast to the permanent ischemia model used in our study, these models comprise a proximal transient occlusion of the MCA which causes extensive lesions and a high mortality rate. In the present study, the majority of experiments was performed in a model of permanent occlusion of the distal MCA, thereby inducing cortical, medium-sized ischemic lesions of approx. 15% of the ipsilateral hemisphere. This lesion resembles embolic infarctions in the MCA territory which are observed in a large portion of patients with ischemic strokes. In contrast to the cited investigators, we did not see a beneficial effect of FTY720 treatment on infarct volume either 3d or 7d after MCAO. Furthermore, it did not matter whether the treatment was started before or after stroke nor whether the drug was given in a single dose or daily or whether the drug was administered per oral gavage or intraperitoneally. We also used a model of extensive focal brain lesions as used in the previous studies and did not detect a reduction in infarct size at 24 h after brain ischemia, neither by oral nor intraperitoneal drug administration.

An advantage of the “MCA coagulation model” is that it allows to study the pathophysiology and potential treatment effects for prolonged periods after stroke in mice because the mortality rate, an important potential confounder, is low (<5%). We observed a substantial reduction of postischemic cerebral lymphocyte influx by FTY720 compatible with reports in studies of primary neuroinflammatory diseases [22]. In contrast, microglia/monocyte counts were not affected by the treatment and granulocyte invasion was even strongly increased in some animals. This apparent heterogeneity of the FTY720 effect on the post-ischemic immune system was also reflected in the expression pattern of cerebral cytokines. Three days after MCAO, cerebral IFN-γ expression - which is mainly produced by invading lymphocytes at this time [13] - was attenuated by FTY720. In contrast, we observed a substantial increase of pro-inflammatory cytokines (e.g IL-1β) 24 h after MCA which are mainly secreted by invading monocytes and activated local microglia [39], [40] and have predominantly harmful effects at this stage [16], [41], [42], [43].

The lack of brain protection despite effective inhibition of lymphocyte transmigration – a therapeutic concept that was efficacious in several other studies [10], [12], [13], [14], [44], [45] – may have alternative explanations. First, in contrast to primary neuroinflammatory diseases, the physical integrity of the blood-brain barrier is severely disturbed after ischemic stroke [46] which may allow uncontrolled brain entrance and potentially cytotoxic effects of FTY720. Effects of FTY720 treatment on oligodendrocytes, astrocytes, oligodendrocyte progenitor cells and neurons have been investigated in several *in vitro* studies, documenting the multiple pathways that are targeted by S1P. Interestingly, neuronal activation, differentiation and neurite outgrowth and retraction by FTY720 seems to be differentially affected under physiological conditions and in an inflammatory milieu that increases the sensitization for S1P signaling [see [47] for detailed review]. Secondly, besides affecting the cellular migration of immune cells, S1P has distinct modulatory functions on their maturation and activation [48]. For example, S1P can change the macrophage phenotype from a pro-inflammatory M1 to a IL-10 secreting M2 phenotype [49], [50]. Thereby, antagonists of S1P-receptors such as FTY720 may prevent a downregulation of innate immune responses after brain ischemia which are commonly observed [51]. Indeed, we detected an increased production of IL-1β and downregulation of IL-10 secretion in brains of FTY720 treated animals. Recently, Michaud et al.[52] proposed a novel mechanism of activation and increased macrophage accumulation at the site of inflammation by S1P-inhibition, opposing to the arrest of lymphocyte-egress. This may correspond to the 3-fold increase of circulating monocytes as early as 6 h after FTY720 administration. Conceivably, FTY720 treatment has on the one side a potent effect on the migratory capabilities of lymphocytes that might be beneficial after ischemic stroke. On the other side the peripheral immune system is substantially altered after acute brain lesions [29], [30], [53] and might be especially susceptible to S1P antagonist-induced paradox effects, like the activation of innate immune cells.

Additionally, previous studies have shown a function of S1P receptors in cardiac function [54] and constriction of cerebral arteries [55]. We therefore investigated the effect of FTY720 treatment on mean arterial pressure and CBF but did not detect significant alterations of these parameters by the treatment.

In conclusion, our study fails to confirm previous reports of a protective effect of FTY720 in experimental ischemic stroke. Besides differences of experimental modeling among studies, our findings suggest a heterogeneity of immunological effects of FTY720 including an enhanced activation of innate immune responses in the early phase after brain ischemia. Establishing immunomodulatory therapies such as FTY720 as a potential treatment for ischemic stroke has to take into account the complexity and context-dependency of the postischemic systemic and cerebral immune modulation.

## Materials and Methods

### Animals

The study was conducted in accordance with national guidelines for the use of experimental animals, and the protocols were approved by the governmental committees (Regierungspraesidium Karlsruhe, Germany). We used age-matched, mature male mice (C57BL/6, 8–10 weeks old, Charles River Laboratories).

### Animal models

Mice were treated daily by oral gavage (p.o.) with 1 mg/kg FTY720 in 100 µl PBS starting at 48 h before or at 3 h after ischemia induction, control animals received PBS. Animals shown in Fig. 2D were treated p.o. with a single dose of FTY720, mice shown in Fig.3C/D were treated with daily intraperitoneal (i.p.) injection of 1 mg/kg FTY720 in 100 µl PBS starting at 48 h before MCAO.

#### *Coagulation model*

We used this model for all experiments as previously described [56], except for data depicted in Fig. 3B and 3D. Briefly, mice were anesthetized with 1.0–2.0% halothane in O2/N2O. After making a 1 cm skin incision between the left eye and ear, a burr hole was drilled through the temporal skull. The dura mater was removed and the middle cerebral artery (MCA) was occluded permanently using a bipolar electrocoagulation forceps (ERBOTOM, Erbe). For laser doppler measurements, we placed the probe (P403, Perimed) 3 mm lateral and 1 mm posterior to the bregma and obtained relative perfusion units (Periflux4001, Perimed). Only animals in which relative CBF dropped below 25% of preischemic baseline after MCAO were included in the analysis. During the operation, body temperature was kept at 37°C using a feedback controlled heating pad. After suturing the skin lesion, the mice were placed in a cage under an infrared heating lamp until recovery from anesthesia. The overall mortality in the coagulation model was less than 5% during the 7d observation period.

#### *Filament model*

We used this model exclusively for experiments shown in Fig. 3. Mice were anesthetized with 1.0–2.0% halothane in O2/N2O-enriched air. We placed the laser doppler probe over the cortical area supplied by the MCA. Baseline CBF was measured as relative perfusion units and defined as 100% flow. After neck dissection, we made an incision into the left external carotid artery (ECA) between two ligations and advanced a silicon-covered 8-0 nylon monofilament through the internal carotid artery to occlude the MCA. MCA occlusion was documented as a decrease in relative perfusion values by laser doppler to less than 20% of original flow, a less pronounced decrease in CBF was used as an exclusion criterion. We fixed the filament in this position by ligation, closed the neck, removed the doppler probe and placed the mouse in its cage. Then, 60 min after filament insertion the mouse was reanesthetized and the filament removed. After we closed the surgical wound, the mice were transferred to their cages with free access to water and food. During the operations body temperature was kept at 37°C with a feedback controlled heating pad. We maintained normal body temperature between operations and until recovery after the procedure by an infrared heating lamp.

A separate group of animals received placement of a laser doppler probe as described above. Additionally, a catheter was placed into the left iliac artery through the femoral artery for consecutive recording of relative cerebral blood flow and arterial blood pressure before and after treatment with FTY720 (1 mg/kg) or PBS.

### Functional outcome tests

For the “forelimb use asymmetry test” [32], we placed the mice in a transparent glass cylinder and videotaped them for 3–10 min, depending on degree of movement. The independent use of the forelimb was analyzed by a video player with slow motion and frame-by-frame capabilities (VLC Media Player). To assess independent use of left or right forelimb we scored (1) contact of the cylinder wall with one forelimb during a full rear and (2) landing with only one forelimb at the cylinder bottom after a full rear. At least 20 independent contacts were counted for one forelimb and each experiment was performed twice, with a 1 h break for the mouse between testing. Forelimb use asymmetry was expressed as a ratio of right and left-sided, independent forelimb use.

For the “corner test” [31], we placed the mice between two boards set at a 30° angle and allowed to move freely. We counted left and right turns with a rearing movement after deep entry in the corner and calculated the ratio of right and left turns as an indicator of behavioural asymmetry. At least 12 full turns after a rearing movement were counted for each testing.

### Assessment of infarct volume

We deeply anesthetized the mice and perfused them transcardially with 20 mL normal saline. We removed the brains from the skull and froze them immediately in isopentane (−20°C). We cut 20 µm thick coronal cryosections every 400 µm, stained the sections using the high-contrast silver staining protocol [57], scanned them at 600 dpi, and analyzed the infarct area on each section (Scion Image). For the permanent coagulation model inducing exclusively cortical infarcts, the Swanson method [58] was applied for indirect infarct measurement and correction for cortical swelling: [Ischemic area]  =  [Cortex area of the contralateral side] – [Non-ischemic cortex area of the ipsilateral side]. For the filament model comprising also subcortical lesions, we used direct infarct measurement and corrected for brain edema by subtracting the ipsilateral minus contralateral hemisphere area from the directly measured infarct area. The total infarct volume was determined by integrating measured areas and distances between sections.

### Brain edema measurement

Anesthetized mice were sacrificed, the brain was removed and each hemisphere was weighed using an electronic analytical balance (Sartorius) to determine the wet weight (WW) [59]. Then, both hemispheres were dried in an oven at 70°C for 48 h and weighed again to obtain the dry weight (DW). The formula (WW - DW) / WW x 100 was used to calculate the water content of each hemisphere as a percentage. The data was expressed as the index of the percentage water content in the ischemic divided by the non-ischemic hemisphere.

### Evans blue assay

Blood brain barrier permeability was measured by the Evans blue method, according to previous reports [60], [61]. Mice were injected i.v. with Evans blue dye and then perfused with PBS 1 h later, Each hemisphere of brains at 3d after MCAO were homogenized and fluorescence was measured on a fluorophotometer (Synergy 4, Biotek) at 680 nm. Fluorescence intensity of ischemic hemispheres was normalized to the values of the respective non-ischemic hemisphere.

### Differential blood cell counting

Blood samples were collected at the respective time points after treatment by retroorbital puncture and immediately analyzed in the Core Laboratory Facilities of the University Hospital Heidelberg for a complete blood cell count.

### Flow cytometry

We collected blood samples and secondary immunological organs at various time points after MCAO for flow cytometric analysis of leukocyte subpopulations. We used a previously published protocol for quantitative FACS analysis and respective gating strategy of leukocyte subpopulations infiltrating the brain [13], [34]. We stained single cell suspensions for anti-mouse CD3 (Clone 17A2), CD4 (Clone RM 4-5), CD25 (Clone 7D4), Foxp3 (Clone FJK-16s), B220 (Clone RA3-6B2), NK1.1 (Clone PK136), Gr-1 (RB6-8C5), CD11b (Clone M1/70), MHC-II (M5/114.15.2) and the appropriate isotype control by following the manufacturer's protocols (eBioscience). We performed flow cytometry on a Becton Dickinson FACS Calibur and analyzed the data by CellQuest Pro software. Gates were set according to unstained samples and isotype control; compensation was adjusted using BD CaliBRITE Beads (BD Bioscience).

### Immunohistology

We performed immunohistochemistry of mature T lymphocytes, B cells and neutrophilic granulocytes on coronal cryostat sections (12 µm), taken at the anatomical landmark of the anterior commissure, after pretreatment with 4% PFA for 60 min. We blocked endogenous peroxidase by Peroxidase Blocking solution (Dako), then incubated the sections with primary antibodies against CD3 (Clone 3H698, Zytomed) or myeloperoxidase (Clone RP-053; Zytomed) for 60 min at 21°C. Immunoreactivity was visualized by a universal immunoenzyme polymer method (Nichirei Biosciences) and sections developed in diaminobenzidine. We investigated the distribution of activated microglia by immunohistochemical staining of coronal cryostat brain sections (12 µm) for IBA1 (Wako). Immunoreactivity was visualized by the avidin-biotin complex method and sections developed in diaminobenzidine. For analysis of cell number, we captured the images on a Zeiss Axiovert 200 M microscope. Blinded samples were evaluated for absolute cell counts by 3 individual analyzers (A.L., W.Z., H.D.) not informed about treatment groups. One complete ischemic hemisphere per mouse was analyzed on coronal sections at the anterior commissure position by consecutive high power field analysis for the presence of positively stained cells. All images for the analysis of IBA1+ cell numbers were processed with the TissueQuest software (TissueGnostics) for cell-based counting of automatically recognized IBA1 positive cells in a FACS-like manner of scattergram analysis [13], [62]. Nonspecifically stained structures and objects that were too large were excluded from the analysis.

### RNA isolation and RT-PCR

We isolated RNA from separated cerebral hemispheres with RNApure (Peqlab) and performed reverse transcription with the High Capacity cDNA Archive Kit (Applied Biosystems) on an ABI7500 RT-PCR System (Applied Biosystems). Primers were purchased as ready-to-use primer sets for each gene (Super Array). All assays were run in duplicate. We normalized the results for each individual gene to the level of the housekeeping gene encoding for peptidylprolyl isomerase A (cyclophilin).

### Cytokine enzyme-linked immunosorbent assay

We collected serum by retroorbital puncture and centrifugation and froze it at −80°C immediately until analysis of cytokine protein concentrations with commercial kits for the quantitative assay of INF-γ, IL-10 (all from R&D Systems), TGF-β, IL-6 and TNF-α (eBioscience). Detection limits were 2.0 pg mL-1 for INF-γ, 4.0 pg mL-1 for IL-10, 7.0 pg mL-1 for TGF-β, 4.0 pg mL-1 for IL-6 and 5.1 pg mL-1 for TNF-α.

### Statistical analysis

All values in bar graphs are expressed as mean ± standard deviation (SD). We analyzed infarct volumes and functional outcome tests by two-tailed Student's t test after validating the normal distribution of these data sets (Kolmogorov-Smirnov test). For the remaining data, we used two-tailed Wilcoxon rank-sum test, using GraphPad Prism 5 software. A p<0.05 was considered statistically significant.
